# Local Microbubble Removal in Polydimethylsiloxane Microchannel by Balancing Negative and Atmospheric Pressures

**DOI:** 10.3390/mi15010037

**Published:** 2023-12-23

**Authors:** Yasunori Tokuoka, Tadashi Ishida

**Affiliations:** Department of Mechanical Engineering, School of Engineering, Institute of Technology, Tokyo 226-8503, Japan

**Keywords:** microfluidics, long-term perfusion, microbubbles, local removal, polydimethylsiloxane

## Abstract

Long-term experiments using organoids and tissues are crucial for drug development. Microfluidic devices have been regularly used in long-term experiments. However, microbubbles often form in these devices, and they may damage and starve cells. A method involving the application of negative pressure has been reported to remove microbubbles from microfluidic devices composed of polydimethylsiloxane; however, negative pressure affects the cells and tissues in microfluidic devices. In this study, a local microbubble removal method was developed using a microfluidic device with 0.5 mm thin polydimethylsiloxane sidewalls. The thin sidewalls counterbalanced the negative and atmospheric pressures, thereby localizing the negative pressure near the negatively pressurized chamber. Microbubbles were removed within 5 mm of the negatively pressurized chamber; however, those in an area 7 mm and more from the chamber were not removed. Using the local removal method, a long-term perfusion test was performed, and no contact was confirmed between the bubbles and the simulated tissue for 72 h.

## 1. Introduction

Microfluidic devices are used to perform biological assays and analyses with precise control of chemical concentration and flow and reveal crucial biological and medical insights [1,2,3,4,5]. Thus, they are commonly used in the biological and medical fields, including for virus detection, genetic disease diagnosis, and drug development [6,7,8,9]. During drug development, cellular responses to drugs are measured using cells cultured in microfluidic devices. Although cells change their signaling pathways and behavior in response to dosed drugs, their responses differ under different cellular conditions. Cells in organoids/tissues have cell–cell and cell–extracellular matrix (ECM) interactions between different cell types and ECMs and express actual biological behaviors in vitro which do not appear in single or monolayered cells [10,11,12,13]. For drug development, biological behavior must be maintained over a long period (e.g., several tens of hours) to study cellular responses to drugs. However, the long-term culture of organoids and tissues is challenging because of culture conditions and waste accumulation. Therefore, perfusion cultures of the organoids and tissues were developed using microfluidic devices [14,15,16,17,18], which enable the expression of actual behaviors and long-term cell culture. However, microbubbles (MBs, optically visible at the micro scale) often form in microfluidic devices, change/stop the flow of culture medium in a microchannel, and apply surface tension to cells when MBs and cells are brought into contact. These influences interfere with long-term perfusion cultures [19,20,21,22], analytical techniques, and biological assays [23,24,25].

Nanobubbles (NBs, optically invisible due to too small size or dissolved in liquid) usually remain in/on the microstructure of a microfluidic channel and form larger MBs and even bubbles (optically visible at the milliscale). Several methods are available for preventing bubble formation. For example, washing channels with a highly hydrophilic liquid, such as ethanol, can remove NBs stuck in the channels [19,20]. However, NBs and MBs accumulate and form bubbles in microchannels during long-term cultures of over 24 h [20]. This is because dissolved gases exist in liquids and frequently adhere to the microfluidic channel surface as MBs due to the high surface-to-volume ratio of the microstructures [23]. This adhesion of MBs to channel walls changes the flow direction in the channel [19]. Furthermore, the adhered bubbles grow via coalescence and occasionally contact organoids and tissues in a perfusion culture. This causes damage to cells in organoids and tissues owing to high surface tension [19]. As a countermeasure against MBs in microfluidic devices, traps that are higher than other microfluidic parts and located upstream are typically used to remove MBs from the culture medium [19,26]. Although traps exist, their capacity for trapping bubbles is limited. Trapped MBs grow, occasionally dropping out of traps during long culture periods and flowing into cell culture areas, thereby damaging cells. Thus, MBs must be removed from microchannels to achieve a long-term perfusion culture for a sufficient duration.

MBs in a culture medium can be removed by applying negative pressure to polydimethylsiloxane (PDMS) microchannel walls, which exhibit high gas permeability [27,28,29]. Essentially, when a negative pressure is applied, the entire device is negatively pressurized through the PDMS structure, and the excess gases dissolved in the culture medium are removed. Simultaneously, cells in the microchannel are negatively pressurized, which affects their cellular activities (e.g., growth and differentiation) [30,31,32,33], and the partial pressures of oxygen and carbon dioxide concentrations around the cells and tissues change. Thus, the culture is adversely affected when the entire channel is exposed to negative pressure [34,35].

In this study, a local MB removal method using a microfluidic device was proposed. Herein, the microchannel was surrounded by thin PDMS sidewalls, and the negatively pressurized PDMS sidewalls were exposed to atmospheric pressure. Thus, the negative and atmospheric pressures were balanced, negative pressure was localized only in the bubble-removal area, and MBs were removed from a limited area for 72 h. The proposed local MB removal method was integrated into microfluidic devices for long-term perfusion cultures. No contact was observed between the bubbles and simulated tissue after 72 h.

## 2. Materials and Methods

### 2.1. Working Principle of the Removal of MBs

**Figure 1:**
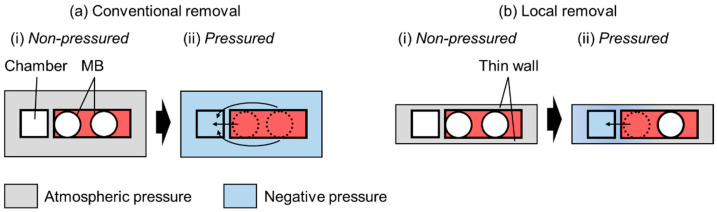
Principle of MB removal. (**a**) Microchannel with conventional removal. (**b**) Microchannel with local removal and thin PDMS sidewalls.

The working principle of MB removal is shown in Figure 1. In conventional removal methods, MBs are initially present in channels with thick sidewalls (Figure 1a(i)). Thus, when negative pressure is applied to the chamber, the PDMS microchannel is negatively pressurized because of the PDMS connection. The PDMS surface in contact with the atmosphere is at atmospheric pressure, whereas the surface in contact with the microchannel is not because of the thick sidewalls. Thus, MBs close to and away from the chamber are removed (Figure 1a(ii)). The removal rate *dV*/*dt* through a thin PDMS wall can be theoretically expressed as follows [15]:

(1) $$-\frac{dV}{dt}=\frac{PA\left(p_{2}-p_{1}\right)}{b}\frac{T}{273}\frac{76}{p_{atm}}$$

where P is the gas permeability of PDMS; *A* is the surface area through which the bubble is removed; *p*_2_ − *p*_1_ is the pressure difference; *b* is the thickness of the thin wall; *T* is the temperature; and *p_atm_* is the atmospheric pressure (cmHg).

In the local MB removal method, the PDMS sidewall of the channel is thin and exposed to the atmosphere (Figure 1b). Thus, MBs are present in the channel (Figure 1b(i)). When negative pressure is applied to the chamber, the pressure in the thin PDMS sidewall can be determined by balancing the negative and atmospheric pressures. Essentially, the pressure outside the chamber is atmospheric, whereas that near the chamber remains negative. Therefore, only the MBs close to the chamber disappear (Figure 1b(ii)).

### 2.2. MB Removal Tests

#### 2.2.1. Microfluidic Device with Local MB Removal

**Figure 2:**
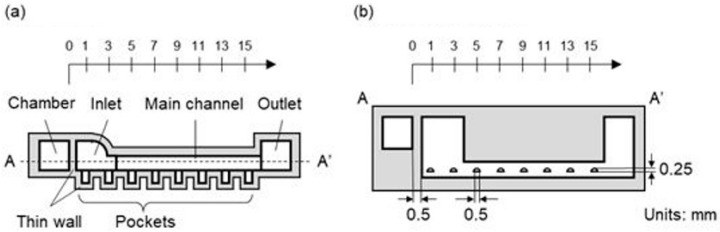
Schematic of the microfluidic device with local MB removal. (**a**) Plan view. (**b**) A-A′ sectional view.

The microfluidic device used for local MB removal is shown in Figure 2 and comprised a main channel, pockets, and a chamber (Figure 2a). The main channel was for liquid perfusion, whereas the pockets were used to form MBs at certain positions. The chamber was subjected to negative pressure. The height and width of the main channel were 1.5 and 1.0 mm, respectively; the pocket was semicylindrical, with a diameter of 0.5 mm and length of 1.0 mm (Figure 2b). The thin wall between the main channel and the chamber and the thin sidewalls of the pockets were all 0.5 mm thick. Pockets were positioned from 1 to 15 mm every 2 mm from the edge of the chamber, which was set as 0 mm. A microfluidic device for conventional MB removal with thick sidewalls was fabricated for comparison with local MB removal. The pockets and chambers of the conventional MB removal microfluidic device had sidewalls with a thickness of >3 mm.

#### 2.2.2. Electrical Circuit Model of Microfluidic Device with Local MB Removal

**Figure 3:**
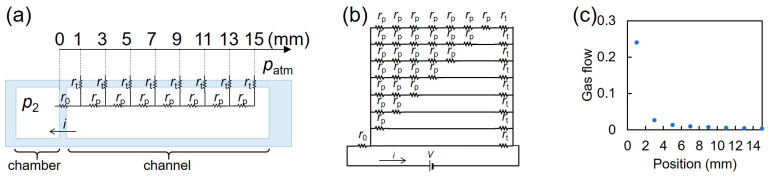
Electric circuit model of local bubble removal. (**a**) Electric circuit model of local bubble removal. (**b**) Equivalent circuit of local bubble removal. (**c**) Gas flow at pockets through PDMS sidewalls as a function of their positions.

We modeled local bubble removal using the electric circuit theory, as shown in Figure 3a. We used the analogy between gas flow through PDMS driven by negative pressure and electric current through resistance driven by voltage. Here, we set the gas flow *i*, the resistance from the chamber to the first pocket *r*_0_, the resistance between pockets *r_p_*, the resistance through sidewalls *r_t_*, and the pressure between the chamber and the atmosphere *V* (=*p*_2_ − *p_atm_*). The equivalent circuit of the local bubble removal setup should have parallel resistance, as shown in Figure 3b. Considering that the distance from the chamber to the first pocket was 1 mm, the distance between pockets was 2 mm, and the thickness of sidewall was 0.5 mm, *r*_0_ = 2*r_t_* and *r_p_* = 4*r_t_*. The total resistance was calculated to be 2.64*r_t_*, resulting in a gas flow of 0.38 *V*/*r_t_*. The pressure to drive the gas flow at 2*k* − 1 mm (*k*th pocket) should be $V_{k}=\frac{0.24V}{4\left(k-1\right)+1}$. With this, the gas flow was calculated to be $\frac{0.24V}{(4\left(k-1\right)+1)r_{t}}$. The gas flow at the 4th pocket was less than 0.01 *V*/*r_t_*, whereas the gas flow at the 1st pocket was 0.24 *V*/*r_t_*, as shown in Figure 3c.

#### 2.2.3. Microfluidic Device for Long-Term Perfusion

**Figure 4:**
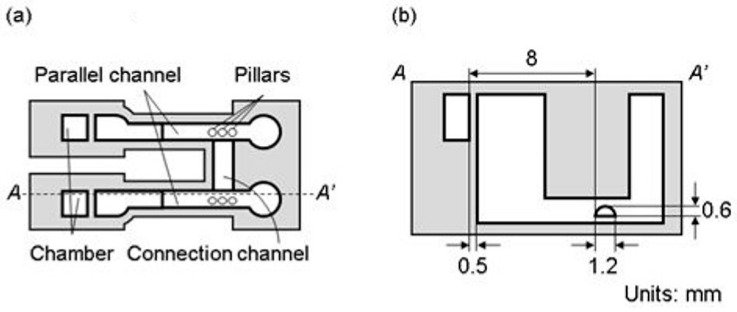
Schematic of microfluidic device for long-term perfusion. (**a**) Plan view. (**b**) A-A′ sectional view.

The microfluidic device for long-term perfusion is shown in Figure 4. To apply the H-shaped channel device for a tissue proposed in Ref. [36], the microfluidic device for long-term perfusion had parallel channels with traps, a connection channel, and chambers (Figure 4a). Parallel and connection channels were used for the perfusion culture medium and set of tissues, respectively. The parallel channels and chambers had the same dimensions as those described in Section 2.2.1. The connection channel possessed a semicylindrical shape, with a diameter of 1.2 mm and length of 3 mm (Figure 4b). The tissue was located 8 mm from the chambers based on the results of the MB removal test. The thin walls between the parallel channels, those between chambers, and the sidewall of the connection channel were all 0.5 mm thick. Additionally, three pillars with a diameter of 0.5 mm and height of 1.0 mm were in the parallel channel to prevent cancer tissue from moving out.

#### 2.2.4. Fabrication Process and Assembly

The microfluidic device was fabricated via PDMS molding and assembly [36] and comprised upper, middle, and lower layers. The molds for each layer were machined (MDX-540, Roland DG, Shizuoka, Japan), and the surface of the mold was polished using sandpaper and an abrasive compound. A mixture of PDMS (Silpot184 W/C, Dow Corning Toray, Tokyo, Japan; main agent and hardener at a mass ratio of 10:1) was de-aerated in a vacuum container, poured into each mold, and de-aerated again at −80 kPaG for 20 min. Furthermore, PDMS was cured at 100 °C for 60 min, and the PDMS replica was demolded.

**Figure 5:**
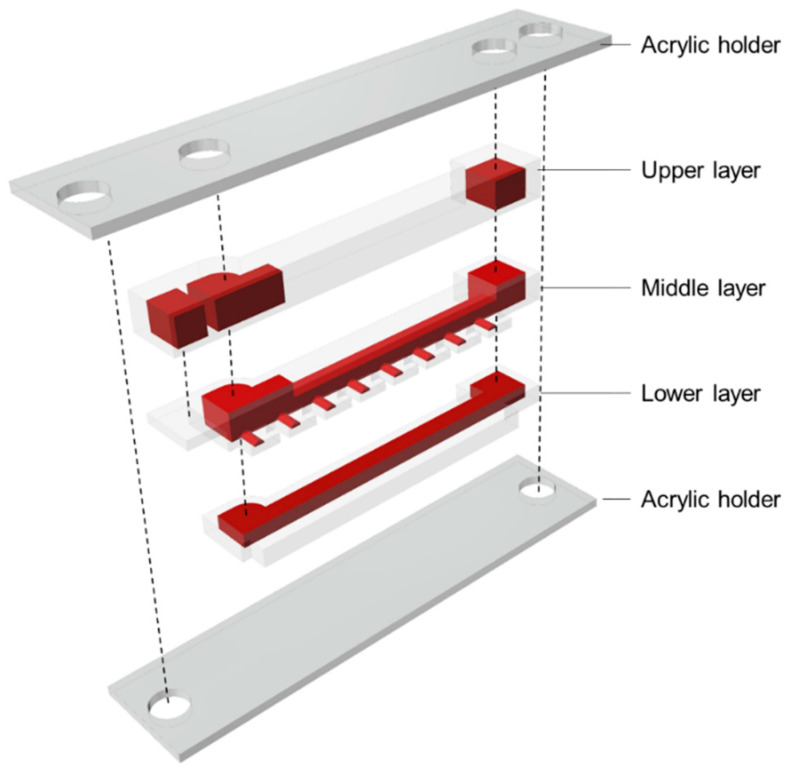
Schematic of the microfluidic device.

The PDMS layers were assembled, as shown in Figure 5. Because each layer of the microfluidic device is convex and concave for alignment, misalignment of each layer could be suppressed. The aligned PDMS layers were pressed and screwed between the acrylic holders without misalignment thanks to the alignment structures.

#### 2.2.5. Experimental Setup

**Figure 6:**
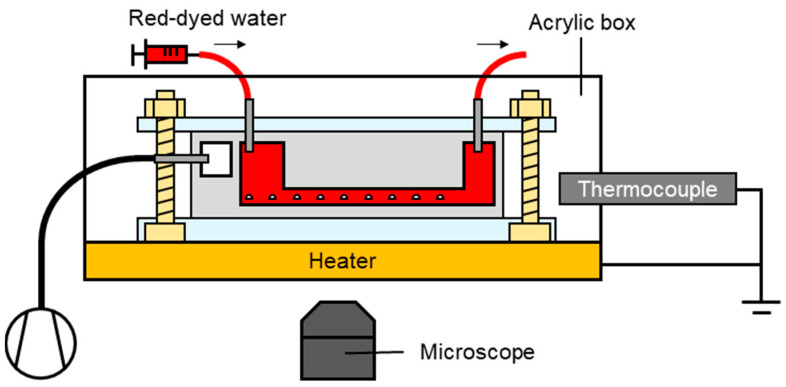
Experimental setup for the MB removal test.

The experimental setup is illustrated in Figure 6. The microfluidic device was sterilized in an autoclave at 127 °C for 30 min. In particular, the microfluidic device in the acrylic box was placed on the stage of an inverted optical microscope (CKX41, Olympus, Tokyo, Japan) and connected to a syringe pump (KDS210, KD Scientific, Holliston, MA, USA) with a silicone tube. The syringe (SS-50ESZ, Terumo, Tokyo, Japan) was filled with red-dyed water and warmed to 37 °C in the incubator, and bubbles were carefully removed manually before the experiment. Red-dyed water was prepared by mixing the food dye (Food Coloring Red, Kyoritsu Foods, Saitama, Japan) with pure water at a ratio of 0.2 g/100 mL.

Red-dyed water flowed in the main channel at 10 μL/min for 72 h. During perfusion, the chamber near the main channel was evacuated using a vacuum pump (VP0940; Nitto Kohki, Tokyo, Japan) down to −50 kPaG. Images of the main channel and pockets inside the microfluidic device were captured every 5 min. The temperature in the acrylic box was adjusted using a thermocouple and heater. In the experiment utilizing the microfluidic device, the parameters were P = 1.92 × 10^–15^ m^2^·s^–1^·Pa^–1^, *A* = 0.39 mm^2^, *p*_2_ − *p*_1_ = −50 kPaG, *b* = 1–15 mm, *T* = 310 K, and *p_atm_* = 76 cmHg.

For the long-term perfusion test, the fluid was changed from red-dyed water to Dulbecco’s modified Eagle medium (DMEM, FUJIFILM Wako Pure Chemical Corporation, Osaka, Japan; Figure 6). The partial pressure in the acrylic box was set to 5% CO_2_.

#### 2.2.6. Calculation of the MB Removal Rate

The MB removal rate was defined as the rate at which red-dyed water entered the pocket. Because the pocket was semicylindrical shape, the rate of red-dyed water entering the pocket was calculated as follows:

(2) $$\frac{\pi d^{2}L}{8t}$$

where *d* is the diameter of pocket (0.5 mm), *L* is the length of the pocket that the red water enters, and *t* is the time. The area of red-dyed water observed at time *A* was calculated as d*L*. Here, *A* was obtained by subtracting the initial condition image from images at each time point using ImageJ 1.54f software. Thus, the rate of red-dyed water entering the pocket was calculated using the above Equation (2).

## 3. Results

### 3.1. Assembled Microfluidic Device

**Figure 7:**
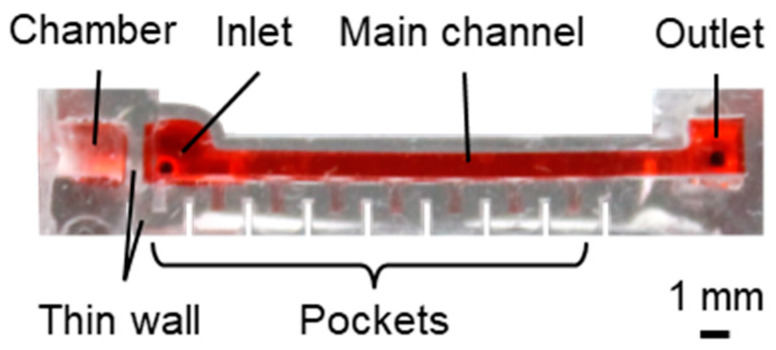
Fabricated microfluidic device with local MB removal.

**Figure 8:**
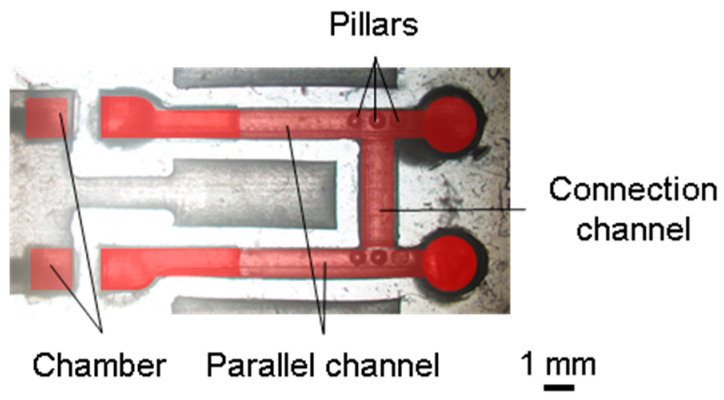
Fabricated microfluidic device for long-term perfusion.

The microfluidic devices assembled for local MB removal and long-term perfusion are shown in Figure 7 and Figure 8, respectively. The sidewalls of the microfluidic devices were 0.5 mm as designed.

### 3.2. Results of the MB Removal Test

**Figure 9:**
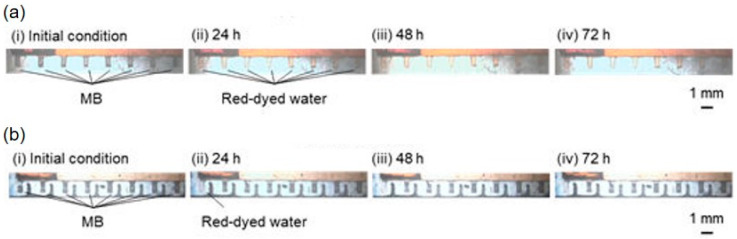
Comparison of MB removal tests using conventional and local removal. (**a**) Conventional removal test. (**b**) Local removal test.

MB removal tests were performed for the cases of the conventional and local removal of MBs from the pockets (Figure 9). Initially, the MBs filled these pockets (Figure 9a(i),b(i)). During conventional removal, the MBs close to the chamber were removed faster, with removal rates of 53.8 ± 16.2 nL/h (mean ± standard deviation) at 1 mm and 15.0 ± 10.0 nL/h at 15 mm. Red-dyed water flowed into the pockets after 24 h (Figure 9a(ii)). After 48 h, the MBs were completely removed from all pockets and replaced with red-dyed water (Figure 9a(iii)). This state was maintained for 72 h (Figure 9a(iv)). During local removal, the MBs at 1 and 3 mm from the chamber were removed at 41.0 ± 25.5 and 11.5 ± 8.6 nL/h, respectively, in 24 h (Figure 9b(ii)). After 48 h, the pockets at 1 and 3 mm were filled with red-dyed water; however, no volume changes in the MBs were measured in the other pockets (Figure 9b(iii)). This state was maintained for 72 h (Figure 9b(iv)).

**Figure 10:**
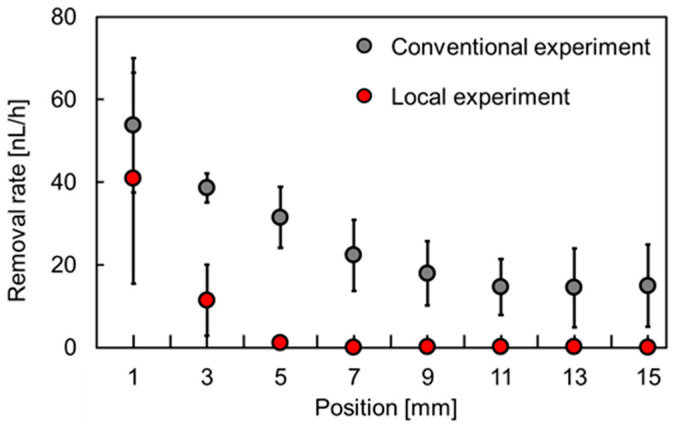
Relationship between the position of the pockets and the rate of MB volume change (N = 3).

Figure 10 shows the decreasing volume rate of MBs as a function of the position of the pockets. Under conventional removal, the removal rate of MBs ranged from 15.0 to 53.8 nL/h, whereas under local removal, the removal rates at 1, 3, and 5 mm were 41.0, 11.5, and 1.2 nL/h, respectively. Essentially, the removal rates for local removal were slightly lower than those for conventional removal. When the sidewalls were thin, the MB removal rate was 0 nL/h at distances greater than or equal to 7 mm from the chamber. In this experiment, pockets within 5 mm of the chamber were under negative pressure, whereas those at distances greater than or equal to 7 mm from the chamber were under atmospheric pressure.

### 3.3. Results of Long-Term Perfusion Test

**Figure 11:**
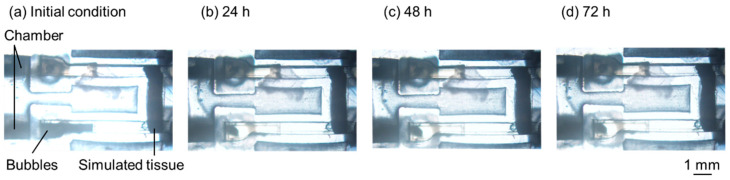
Long-term perfusion test of simulated tissue. (**a**) Initial condition. (**b**) For 24 h. (**c**) For 48 h. (**d**) For 72 h.

A long-term perfusion test was performed to confirm that the bubbles were not in contact with the simulated tissue. The results are shown in Figure 11, where the dark parts in the inlets under the initial condition represent the shadows of the bubbles (Figure 11a). Evidently, after 24 h, the bubbles at the inlet were gradually removed (Figure 11b), and this state was maintained for 72 h (Figure 11c,d). During perfusion, the bubbles did not contact the simulated tissue for 72 h.

## 4. Discussion

A comparison of the conventional and local MB removal methods reveals that the thin wall reduced the MB removal rate by 24, 70, 96, and 100% at 1, 3, 5, and 7 mm from the chamber, respectively. In the pocket located 1 mm from the chamber, the rate difference between conventional and local removal was small because a large negative pressure was applied to both pockets. However, the difference between them was larger because of the atmospheric pressure within the thin wall in the pockets, which were 3 mm away from the chamber.

**Figure 12:**
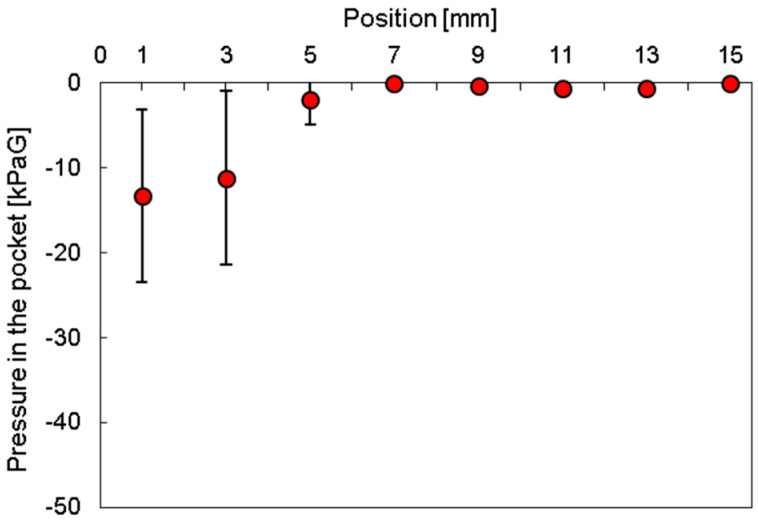
Relationship between the position of the pockets and gauge pressure (N = 3).

Gauge pressures at each position were calculated using Equation (1). Figure 12 shows the relationship between the position of the pockets and the gauge pressure. Evidently, the gauge pressure was −13, −11, −2, and 0 kPaG at 1, 3, 5, and ≥7 mm, respectively. These results suggested that the distances 7 mm and more from the chamber were under atmospheric pressure.

Some studies have reported the use of negative pressure to remove bubbles from a PDMS microchannel [27,28,29]. However, in these reports, compared with this study, the PDMS sidewalls were thicker, and a higher negative pressure was applied to the microfluidic device. Therefore, a negative pressure was applied to the cell culture area. This may affect cell activity and partial pressures around cells and tissues, such as those of oxygen and carbon dioxide [30,32]. However, this method can remove bubbles locally by thinning the sidewalls while maintaining partial pressure. Additionally, this method can realize the long-term perfusion culture of cells and tissues and is a powerful tool for clarifying various biological phenomena.

## 5. Conclusions

In this study, a PDMS microfluidic device was developed for local MB removal. Essentially, the negative pressure in the PDMS is canceled by the inflow of atmospheric air, which is realized by thinning the PDMS sidewalls of the channel in the area at a certain distance from the negative pressure source. In this experimental setup, the MBs close to the chamber disappeared from the microfluidic channel, whereas the MBs located at ≥7 mm from the chamber were not removed. This local MB removal method was applied to an H-shaped microfluidic device, and perfusion was tested for 72 h. Although bubbles were occasionally observed in the inlet, no contact between the bubbles and simulated tissue was observed for 72 h. In the future, we plan to culture living tissues using the microfluidic device for long-term perfusion.

## Data Availability

Data are contained within the article.
